# Tsunamis caused by submarine slope failures along western Great Bahama Bank

**DOI:** 10.1038/srep35925

**Published:** 2016-11-04

**Authors:** Jara S.D. Schnyder, Gregor P. Eberli, James T. Kirby, Fengyan Shi, Babak Tehranirad, Thierry Mulder, Emmanuelle Ducassou, Dierk Hebbeln, Paul Wintersteller

**Affiliations:** 1 CSL-Center for Carbonate Research, University of Miami, 4600 Rickenbacker Cswy., 33149 Miami, USA; 2 Center for Applied Coastal Research, University of Delaware, Newark, DE 19716, USA; 3 UMR 5805 EPOC, Université de Bordeaux, 33615 Pessac Cedex, France; 4 MARUM-Center for Marine Environmental Sciences, University of Bremen, Bremen, Germany

## Abstract

Submarine slope failures are a likely cause for tsunami generation along the East Coast of the United States. Among potential source areas for such tsunamis are submarine landslides and margin collapses of Bahamian platforms. Numerical models of past events, which have been identified using high-resolution multibeam bathymetric data, reveal possible tsunami impact on Bimini, the Florida Keys, and northern Cuba. Tsunamis caused by slope failures with terminal landslide velocity of 20 ms^−1^ will either dissipate while traveling through the Straits of Florida, or generate a maximum wave of 1.5 m at the Florida coast. Modeling a worst-case scenario with a calculated terminal landslide velocity generates a wave of 4.5 m height. The modeled margin collapse in southwestern Great Bahama Bank potentially has a high impact on northern Cuba, with wave heights between 3.3 to 9.5 m depending on the collapse velocity. The short distance and travel time from the source areas to densely populated coastal areas would make the Florida Keys and Miami vulnerable to such low-probability but high-impact events.

## Tsunami threat along florida coastline

Seismic activity along the East Coast of the United States is low, and therefore a potential tsunami risk through seafloor displacements is minimal. There is the possible impact of a tsunami generated in the far field from the flank collapse along the Canary Island of La Palma^1^,^2^,^3^,^4^. Submarine slope failures along the Atlantic Ocean continental margin, however, pose a potential source of tsunami hazard for the highly populated coastal areas of the US East Coast. The 1929 Grand Banks landslide tsunami is an example where a submarine landslide close to the US East Coast caused significant damage^5^,^7^. A tsunami in northern Cuba in 1939 is associated with an earthquake of magnitude *M*_*s*_ = 5.6, along the Nortecubana Fault^8^,^9^. In 2014, three earthquakes with magnitudes of 5.1, 4.7, and 4.1 occurred along the same fault system and illustrate the potential of seismicity close to Florida and the Bahamas.

**Figure 1:**
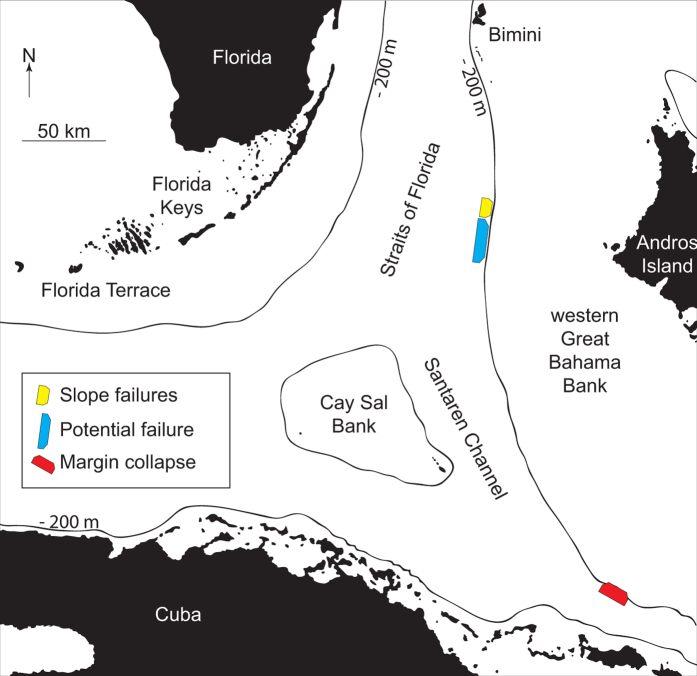
Location of tsunami source areas in the study area (Map was created in MATLAB r2014a using GEBCO grids, http://www.gebco.net).

Failures of the steep slopes of the Bahamian archipelago are possible sources for tsunami-genesis with potential impact on Florida, the Bahamas, and Cuba. Progradation of Great Bahama Bank (GBB) has steepened the slopes of the carbonate platform considerably^10^,^11^. The modern bank morphology consists, from the margin to the basin, of a 100–180 m high escarpment with angles between 25° −70° degrees, slopes of 7–8° declivity in the upper part, and a gradual decrease to the basin floor at approximately 800 m water depth. These slopes are prone to failure even on their lower extents with angles as low as 3°, as identified by multiple past events in the stratigraphic record^11^,^12^,^13^,^14^. In addition, large margin collapses, rock falls, and avalanches occur in southwestern part of GBB, close to the Cuban fold-and-thrust-belt^15^,^16^,^17^. In this study we use volumes of past submarine landslides and a margin collapse along western GBB, which were identified in high-resolution multibeam bathymetric data, sub-bottom profiles and seismic data, to model tsunami impacts along the Florida coastline and northern Cuba (Fig. 1).

## Materials and Methods

To estimate tsunami wave impact on Florida’s eastern coastline and northern Cuba, we use two numerical models in a sequential approach. The non-hydrostatic terrain-following sigma-coordinate model NHWAVE models the initial wave that is generated by the submarine landslide^18^,^19^,^20^. The wave model result provides initial conditions for the next modeling step, the Boussinesq model FUNWAVE-TVD, which models wave propagation using a 500 × 500 m GEBCO grid (General Bathymetric Chart of the Oceans)^21^,^22^,^23^. The GEBCO grid resolution for this purpose is sufficient, since detailed inundation assessment is beyond the scope of this study. Input parameters for the two models were chosen based on the nature and geometry of the landslides identified during morphobathymetric analysis.

### Morphobathymetry

The high-resolution bathymetry and seismic data of the slope failure sites along western slope of GBB were collected during two cruises. During the CARAMBAR research cruise on RV Le Suroît in 2010, a Kongsberg EM302 was used to collect the multibeam echosounder data and a grid of 2D high-resolution multichannel seismic reflection data were shot using a mini-GI 24/24 air gun, and a 96-traces/700-m-long streamer. The CARAMBAR cruise was part of the “Actions Marges” program by the French “Institut National des Sciences de l’Univers (INSU)”, and the University of Bordeaux. Additional high-resolution subbottom profiles (Atlas Parasound™) have been collected with a hull-mounted system on RV Maria S. Merian in 2012 (MARUM, Bremen); these were processed and displayed in Reflex™ v.6. The data for the margin collapse site in southern GBB was provided by the Bahamas Petroleum Company (BPC) and included bathymetry data collected with a Reson SeaBat 8160 59 kHz Multibeam Echosounder (MBES) system and high-resolution single-channel seismic survey acquired using a GeoPulse 5430A sub-bottom profiler system that had a dominant frequency of 3.5 kHz. To analyze bathymetry, the processed multibeam data in ASCII xyz-format was used to produce a 30 × 30 m grid using standard awk (pattern-scanning processing language) and Generic Mapping Tools-GMT v.4.5.9. The resultant digital elevation model (DEM) was visualized with Global Mapper™ v.12 and QPS Fledermaus™ v.7 software. From the DEM, we extracted the landslide parameters such as landslide height (b), length (T), width (w), and slope angle, which were used as input parameters for the landslides in the numerical model. Landslide width is the extent of the scar parallel to the strike of the slope, whereas length is the extent orthogonal to slope strike. Height of the failure scar was measured along the different failure scars from the maximum slope break to bottom of the failure surface; ten profiles across the failure scar were measured and an average value was calculated. Water depth for the failure scar was measured from the top of the scar, but landslide location is implemented in the bathymetry with coordinates and does not have to be specified for the modeling. Sub-bottom profiles provided information about the height of the vertical incision along the incipient failure scar, while the seismic data provided the thickness of the landslide deposit^13^. An overview of the parameters is given in Table 1.

### Numerical models and setup

The submarine slope failure and resulting initial tsunami wave were modeled using the fully dispersive non-hydrostatic wave model NHWAVE 1.1^18^,^19^,^20^. NHWAVE solves either Euler or RANS equations in a surface- and terrain- following sigma coordinate system. We converted the 30 × 30 m resolution grid into UTM and re-gridded the data in MATLAB for implementation in NHWAVE in a 500 × 500 m grid. The initial tsunami wave was then simulated depending on identified source area, landslide scenario, and terminal landslide velocity. The failure body was idealized as smoothed streamlined Gaussian-shaped body. Terminal landslide volume and terminal velocity was calculated according to Enet and Grilli, (2007) using parameters in Table 1^24^. For the landslide volume (*V*_*b*_) we used the approximation of the Gaussian streamlined body: 
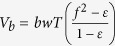
 where *b, w*, and *T* are height, width, and length, respectively, and 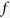, C, and 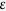 are geometrical factors that are calculated according to 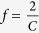 a tan 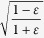, where 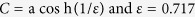 is an experimentally determined parameter^24^. For terminal landslide velocity (*u*_*t*_) we used: 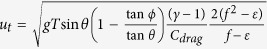 where *θ* = slope angle Cdrag = 1.0, and 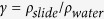. Compared to inertia, gravity, and hydrodynamic forces, the basal Coulomb friction is negligible when the landslide is in motion, which is expressed by assuming
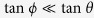
24. The parameters investigated by Enet and Grilli (2007) were recommended as benchmarks for simulating landslide-generated tsunamis during the National Tsunami Hazard Mitigation Program workshop (Galveston, TX, 2010)^20^. The density of the slide material was set to be 1.8 kg/km^3^ based on measurements in the top 150 m of Leg 166 core data^25^.

As modeling results strongly depend on terminal landslide velocity, we performed a series of modeling runs with different velocities and present two end-members here. In one run, we used the method of Enet and Grilli (2007) to calculate terminal landslide velocity based on the parameters derived from bathymetry^24^. Those calculated velocities are higher than velocities measured during the Grand Banks event and a second landslide in Taiwan, where values between 20 and 25 ms^−1^ for 0.5° and 2° slopes, respectively, were observed^7^,^26^. The calculated velocities are thus considered the worst-case scenario, while the 20 ms^−1^ velocity is the more likely scenario. The resulting maximum water surface elevation and velocities from these first simulations then were re-interpolated as input into the fully nonlinear, for Cartesian coordinates fully dispersive Boussinesq model FUNWAVE-TVD 2.0 to simulate wave propagation and estimate an impact at the coastline^21^,^22^,^23^. The model domain includes 864 × 425 grid points in a horizontal Cartesian grid. Three sigma-layers were used in NHWAVE, while FUNWAVE-TVD describes a depth-integrated flow field. FUNWAVE-TVD and NHWAVE are written in Fortran with C-preprocessors, and use OpenMPI as the basis for parallelization. Both codes have been validated for landslide tsunami generation and propagation^23^,^24^,^25^,^26^,^27^,^28^. The code FUNWAVE-TVD is available from https://urldefense.proofpoint.com/v2/url?u=https-3A__github.com_fengyanshi_FUNWAVE-2DTVD_&d=DQICaQ&c=y2w-uYmhgFWijp_IQN0DhA&r=BkBYOpHzQ0vATPH-8yaXkVFyY_4IXuJRBQOZ8IHZzYo&m=QOe25Qe8eevvEQDk8zGdfHORsAjOWLNqtSkbIpLoHzo&s=LgFKNvSEW8a2z8kBlr1CycLca5c48768mdxdqhZhBgs&e=NHWAVE v1.1., used here, is available by request from kirby@udel.edu. The output files were prepared and visualized with MATLAB.

### Model Scenarios And Landslides

**Figure 2:**
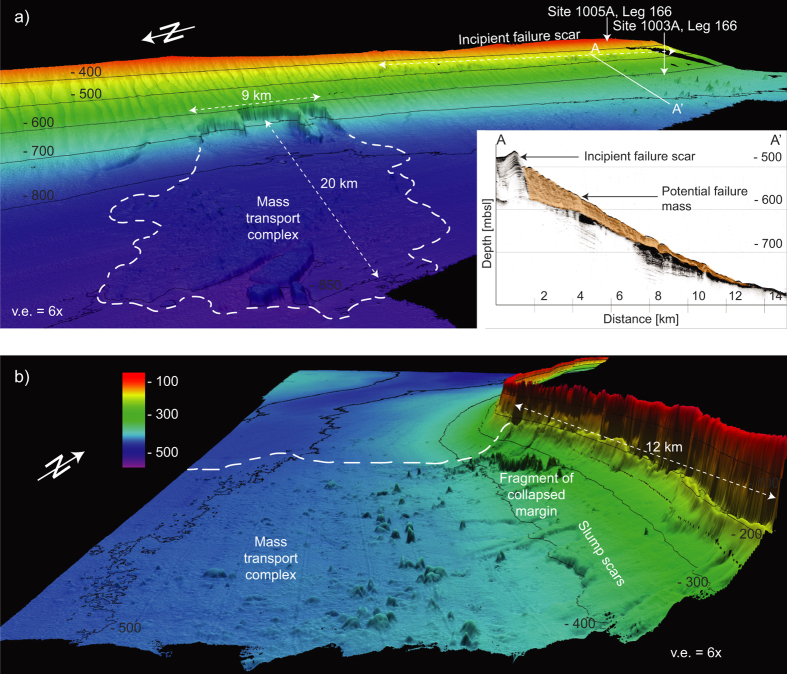
(**a**) Slope failures along western Great Bahama Bank. The scar visible on the lower slope and the associated mass transport complex in the basin. The smaller landslide was simulated using the extent of the middle part of the failure scar. The incipient failure scar (IFS), transect A-A’ is also visible in the subbottom profile. (**b**) Margin collapse and the associated mass wasting products on the southwest corner of GBB. (The 3D bathymetry was produced using QPS Fledermaus v.7 http://www.qps.nl/display/fledermaus/main;jsessionid = C177527398736B33CF4E7113060481F5. The subbottom profle was produced using Reflex v.6 (http://www.sandmeier-geo.de/reflexw.html).

**Figure 3:**
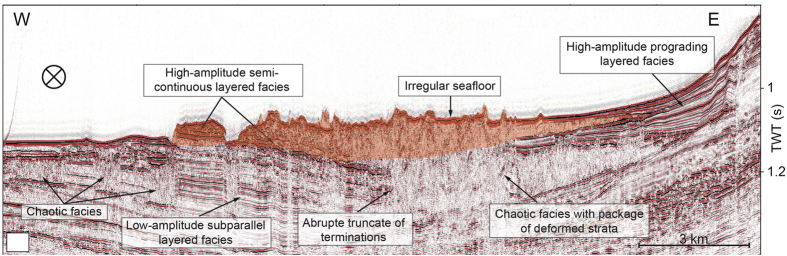
Seismic profile through the MTC. Extent of failure deposit outlined in orange. Modified from Principaud *et al*.^13^.

The four modeled tsunami sources are a submarine landslide on the middle slope modeled as one entity and as a partial failure, a theoretical future landslide indicated by an incipient failure scar on the middle slope, and a margin collapse of the steep carbonate platform in southwestern GBB (Table 1 and Fig. 2). The first model scenario estimates the tsunamigenic potential of a Pleistocene submarine slope failure along the western slope of GBB as one single event, as interpreted by Principaud *et al*.^13^. The Pleistocene mass failure left three failure scars in 600 m water depth with 1.5 to 3 km length, separated by thin elongated spur escarpments, with a total extent of 9 × 3.5 × 0.15 km and a volume of 1.41 km^3^ (Table 1). Additionally, we investigate the effect of a failure scenario where the landslide did not occur as single event, but rather as a multi-stage failure of three independent smaller landslides. For this partial landslide we use the landslide mass from the middle part of the failure scar: 3.7 × 3 × 0.15 km, referred to as partial landslide in Table 1 (Table 1 and Fig. 2a). Sediment mass mobilized or eroded during the landslide on lower parts of the slope is not considered in the model. The calculated terminal landslide velocities are 38.7 ms^−1^ and 35.8 ms^−1^ for the partial landslide. Failure plane inclination is planar and parallel to the adjacent intact slope and limited to confined strata. Its associated mass transport complex (MTC) propagates up to 20 km from the source area^13^,^16^. There is evidence for a partially rigid nature of the landslide; the outer fringe of the MTC consists of disintegrated blocks and the main slide mass is imaged by high amplitude semi-continuous reflections (Fig. 3)^13^. We therefore chose a numerical model using rigid landslide propagation, which can yield results comparable to viscous flow models in order to simulate initial wave height^23^. Hydroplaning on the smooth lower slope and basin might explain the MTC’s large propagation distance^27^. The landslide was stopped by a mounded drift deposit in the basin^13^.

The third scenario models a potential future landslide of a portion of an 80 km long incipient scar on the slope of GBB, between 430 and 450 m water depth (Fig. 2). The scar height is up to 50 m above the seafloor, but the sub-bottom profiles across the incipient scar show up to an 80 m scar incision (Fig. 2). The scar terminates on a high amplitude reflection that is correlated to a cemented lowstand package at 85 m depth. This surface can act as a decollement surface in a future slope failure. The sediment package downslope of the scar shows creeping and slumping features. How wide the failure would be can only be estimated based on the geometry of older failures. Assuming that the incipient scar fails along half of its length, a scenario is modeled using a slide of 40 × 6 × 0.08 km extent along the predefined layer in 80 m depth. The terminal landslide velocity is again chosen as 20 ms^−1^ and for the worst-case is calculated as 48.32 ms^−1^.

The fourth model scenario estimates the tsunami potential of a Pleistocene margin failure observed in southwestern GBB^17^. The top of the margin retreat is in 60 m water depth, has a 12 × 7 × 0.35 km extent and the calculated terminal landslide velocity is 60.25 ms^−1^ (Fig. 2b). Although we model only 12 km of margin collapse, the extent of the collapse might be up to 21 km wide, according to the embayment of the carbonate platform^17^. Collapses of comparable extent are observed along the West Florida margin^17^,^28^.

### Preconditions For Failure

The causes for the slope failure and margin collapse along the Bahamian archipelago are likely inherent in the depositional and diagenetic evolution of the slopes. High sedimentation rates of up to 10 m/k.y. during sea level highstand contribute to the buildup of pore-fluid overpressure in the sediment highstand wedge, resulting in lower shear strength, a common cause for slope failure^28^,^29^,^30^. During sea level lowstands, reduced sediment export of fine-grained platform material results in slightly coarser-grained lowstand layers, which are prone to lithification due to larger grain size and pore water circulation^10^,^11^,^29^. High-frequency sea-level changes produce highstand-lowstand cycles of sediment layers with alternating shear strength^25^. This scenario is described in the glacial-interglacial controlled weak layer theory, where failures preferably occur along lithologically controlled predefined strata^3^,^29^,^30^. Calculations of the Factor of Safety (FS, the ratio of shear strength in relation to effective stress and slope inclination) for the slope sediments, based on shear strength and density in Site 1003A measured during ODP cruise Leg 166 with a Wykeham-Farrance motorized vane shear apparatus^25^, show a FS < 1 below 45 m sediment depth, an indication of an unstable area (Supplementary Fig. S1)^25^,^31^. However, the quality of shear strength measurements might be problematic and, unfortunately, no recent geotechnical data is available for the study area. Values of FS between 1.31 and 2 above 45 m imply moderately to probably stable slopes^31^. Deeper, between 45 and 100 m, where the observed instabilities occur, much lower FS were calculated, with a considerable amount of values lower than 0.7 (extremely and highly unstable)^31^. Seismicity and consecutive lowering of shear strength due to shaking may be the final triggering mechanism for the observed landslides.

### Tsunamis Generated By Submarine Landslides

**Figure 4:**
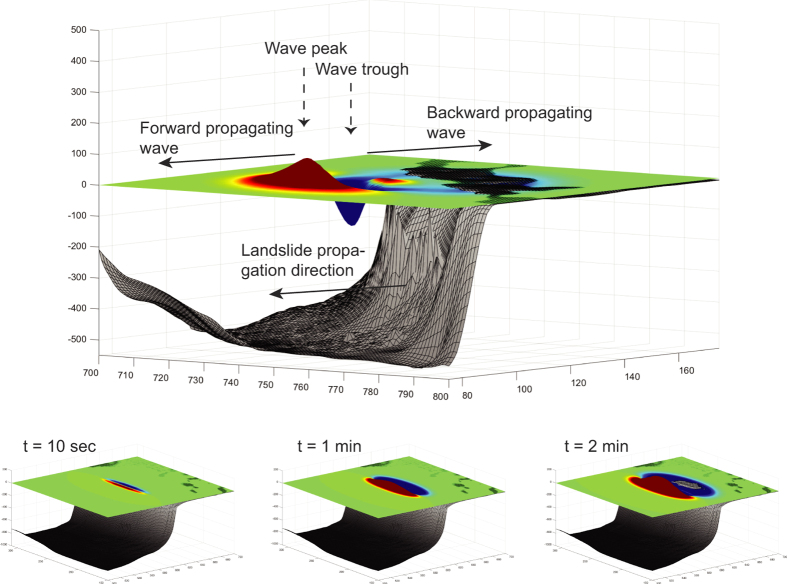
Oblique view through an initial tsunami wave, landslide propagation direction, and wave propagation directions of a symbolic tsunami, and sequence of initial wave generation after 10 sec, 1 min, and 2 min. Not to scale. (Figure was created in MATLAB r2014a using a bathymetric grid acquired during CARAMBAR).

**Figure 5:**
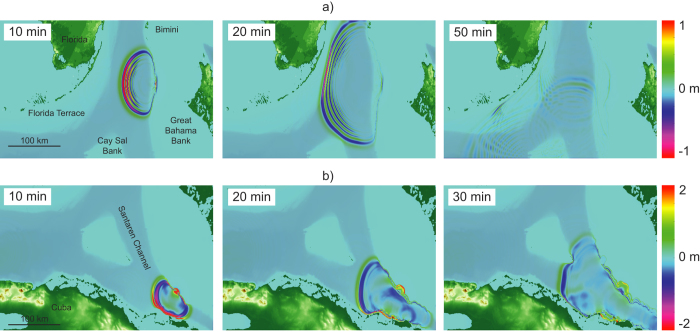
Tsunami wave propagation paths caused by (**a**) a submarine landslide (9 km width and u_t_ = 20 ms^−1^) and (**b**) a margin collapse of southwestern GBB (12 km width and u_t_ = 20 ms^−1^). (Map was created in MATLAB r2014a using GEBCO grids, http://www.gebco.net).

The modeling results for the submarine landslides and the margin collapse along western GBB indicate the generation of tsunamis with destructive potential in the near-source area (Fig. 4). A negative elevation wave followed by a crest propagates backwards onto the bank-top of GBB (Figs 4 and 5a). Once arriving at the platform margin, the back propagating part of the tsunami undergoes both shoaling and reflection due to the shallow water depth (7–20 m), and does not pose a risk for the Bahamian coastline. The main energy of the wave travels as multiple wave trains in the direction of landslide motion. For the source areas on the western slope, the first impact areas are the islands of Bimini. Wave shoaling occurs along the Florida Terrace, where wave speed decreases and amplitude increases. There, a substantial loss of wave energy takes place due to bottom friction^4^,^5^. The first impact wave at the Florida coastline and in the Florida Keys reaches shore after a relatively short propagation time between 35 and 50 min, depending on scenario. The modeled tsunami from the margin failure in southwestern GBB impacts northernmost Cuba after 10 min, and the mainland after only 20 min propagation time, respectively (Fig. 5b). Short wave periods between 60 and 200 seconds were calculated. The wave periods increase for larger and faster landslides.

Estimates of coastal wave heights vary for the different scenarios due to different landslide extent and terminal landslide velocity. For the partial landslide scenario with 20 ms^−1^ terminal landslide velocity, the initial wave height reaches 3.2 m and dissipates quickly during propagation. For the worst-case scenario with 35.8 ms^−1^ terminal landslide velocity, the maximum wave height at coastal impact is 0.3 m. A landslide of such an extent does not pose a threat to the inhabited coastline.

In contrast, the single-event landslide scenario generates initial wave heights of 6.2 m, and reaches the coastline with around 0.3 m wave height after 50 min propagation. If landslide terminal velocity reaches 38.7 ms^−1^ for the worst-case scenario, an initial wave height of 10.9 m is generated, and final impact heights reach between 0.5 and 1 m with wave periods around 70 sec, which would result in dangerous conditions along the coastline.

**Figure 6:**
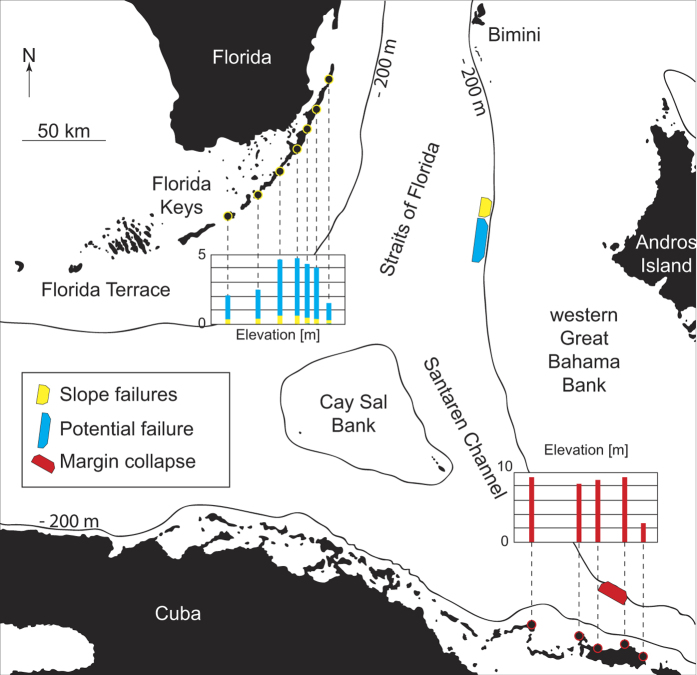
Impact wave height for the worst-case scenarios (calculated terminal landslide velocities) for the slope failure (at 55 min), the potential failure (at 35 min), and the margin collapse (at 20 min). (Map was created in MATLAB r2014a using GEBCO grids, http://www.gebco.net).

The potential slope failure of 40 km of the incipient failure scar with 20 ms^−1^ velocity could generate initial wave heights of 6.3 m, with impact wave heights of around 1.5 m along the coastline. Using the calculated landslide velocity generates an initial wave of 17.2 m height and between 4.5 and 6.7 m impact waves after 30–40 min propagation time (Fig. 6).

The coastline of northern Cuba is only 30 km away from the southeastern GBB margin collapse. A massive collapse as described by Jo *et al*.^17^ results in an initial wave of 20.8 m height and final impact heights of around 3.3 m after only 20 min of propagation with a wave period of 100 sec. The worst-case scenario shows that the first tsunami waves would reach the northernmost coastline of Cuba after only 10 min of propagation with a height of 22.5 m, wave period 200 sec, and impacts major parts of the coast after 20 min with a 9.5 m wave (Fig. 6). Peak wave height maps for all the scenarios are included online (Supplementary Fig. S2).

### Sedimentary Record Of Tsunamis

According to previous studies, the East Coast of Florida is considered threatened by a tsunami, but tsunami deposits have not been found^3^,^5^,^6^. However, the regular occurrence of hurricanes and associated storm surge, with resulting reworking of sediment deposits, probably would mask earlier tsunami-related deposits. Although a tsunami occurred in northern Cuba in 1939, no tsunami deposits are reported there either^8^,^9^. The same fault system associated with the 1939 tsunami showed seismic activity in 2014 with maximum magnitude of Ms 5. Considering the instability predisposing sediment factors discussed above, it is possible that a future strong earthquake can trigger the submarine landslide along the incipient scar observed along GBB (Figs 1 and 2).

## Conclusions

In this study, we use numerical models to reconstruct the tsunamis generated by submarine landslides along western GBB with impact on Florida, and by a margin collapse in southeastern GBB with northern Cuba as main impact area. We further use sediment parameters along GBB to investigate the potential for future landslides. Based on these data and multibeam imagery we identified an area for a possible future landslide.

Landslides modeled with a velocity of 20 ms^−1^ and a few cubic kilometers of volume (1.41–5.53 km^3^) result in final wave impacts of 0.5–1.5 m, which can generate dangerous currents on the coast but do not have the potential for dangerous tsunami waves. However, worst-case scenarios of larger and faster landslides would yield 4.5 m and 9.5 m for the East Coast of Florida and northern Cuba, respectively. The short time between landslide event and tsunami impact poses an important challenge for hazard mitigation. Geotechnical measurements available in the area along with multibeam imagery from older landslides indicate inherent instability of the platform margin and slope that together with the recent seismic activity may trigger a future landslide of larger extent.

## Additional Information

**How to cite this article**: Schnyder, J. S.D. *et al*. Tsunamis caused by submarine slope failures along western Great Bahama Bank. *Sci. Rep.* **6**, 35925; doi: 10.1038/srep35925 (2016).

**Publisher’s note:** Springer Nature remains neutral with regard to jurisdictional claims in published maps and institutional affiliations.

## Supplementary Material

Supplementary Information

Supplementary Information

Supplementary Information

Supplementary Information

Supplementary Information

## Figures and Tables

**Table 1:** Parameters used as input values for tsunami simulation.

	**Parameters of landslides and margin collapse**			
	**Partial landslide (small)**	**Single landslide (large)**	**Potential future landslide**	**Margin collapse**
Height b [m]	150	150	80	350
Width w [m]	3,700	9,000	40,000	12,000
Length T [m]	3,500	3,500	6,000	7,000
Slope [deg]	3.3°	3.3°	3°	4°
Outrun angle*	−180	−180	−180	−130
Depth [mbsl]	600	600	430–450	60
Volume [km^3^]	0.50	1.41	5.73	8.77
Terminal landslide velocity [ms^−1^]	20, 35.8**	20, 38.7**	20, 48.32**	20, 60.25**

^*^Direction of landslide counterclockwise from East.

^**^Calculated for worst-case scenario.
