# Supplementary material for: Tsunamis caused by submarine slope failures along western Great Bahama Bank
Source: Sci Rep. 2016 Nov 4;6:35925. doi: 10.1038/srep35925 (PMC5095707; doi:10.1038/srep35925)

Supplementary Material - Tsunamis caused by submarine slope failures along western Great Bahama Bank

Jara S.D. Schnyder[1,*], Gregor P. Eberli[1], James T. Kirby[2], Fengyan Shi[2], Babak Tehranirad[2], Thierry Mulder[3], Emmanuelle Ducassou[3], Dierk Hebbeln[4], Paul Wintersteller[4]

[1] *CSL-Center for Carbonate Research, University of Miami, 4600 Rickenbacker Cswy., 33149 Miami, USA.*

[2] *Center for Applied Coastal Research, University of Delaware, Newark, DE 19718, USA.*

[3] *UMR 5805 EPOC, Université de Bordeaux, 33615 Pessac Cedex, France.*

[4] *MARUM-Center for Marine Environmental Sciences, University of Bremen, Bremen, Germany.*

***jschnyder@rsmas.miami.edu**

Video V1: Movie of tsunami propagation for the partial landslide scenario. (Movie was created in MATLAB r2014a using GEBCO grids, http://www.gebco.net).

Video V2: Movie of tsunami propagation for the single landslide scenario. (Movie was created in MATLAB r2014a using GEBCO grids, http://www.gebco.net).

Video V3: Movie of tsunami propagation for the potential future landslide scenario. (Movie was created in MATLAB r2014a using GEBCO grids, http://www.gebco.net).

Video V4: Movie of tsunami propagation for the margin failure scenario. (Movie was created in MATLAB r2014a using GEBCO grids, http://www.gebco.net).

Figure S1: Factor of safety, p-wave velocity, and lithologic column for sites 1003A and 1005A from IODP Leg 166. Data was obtained from Eberli et al., (1997).

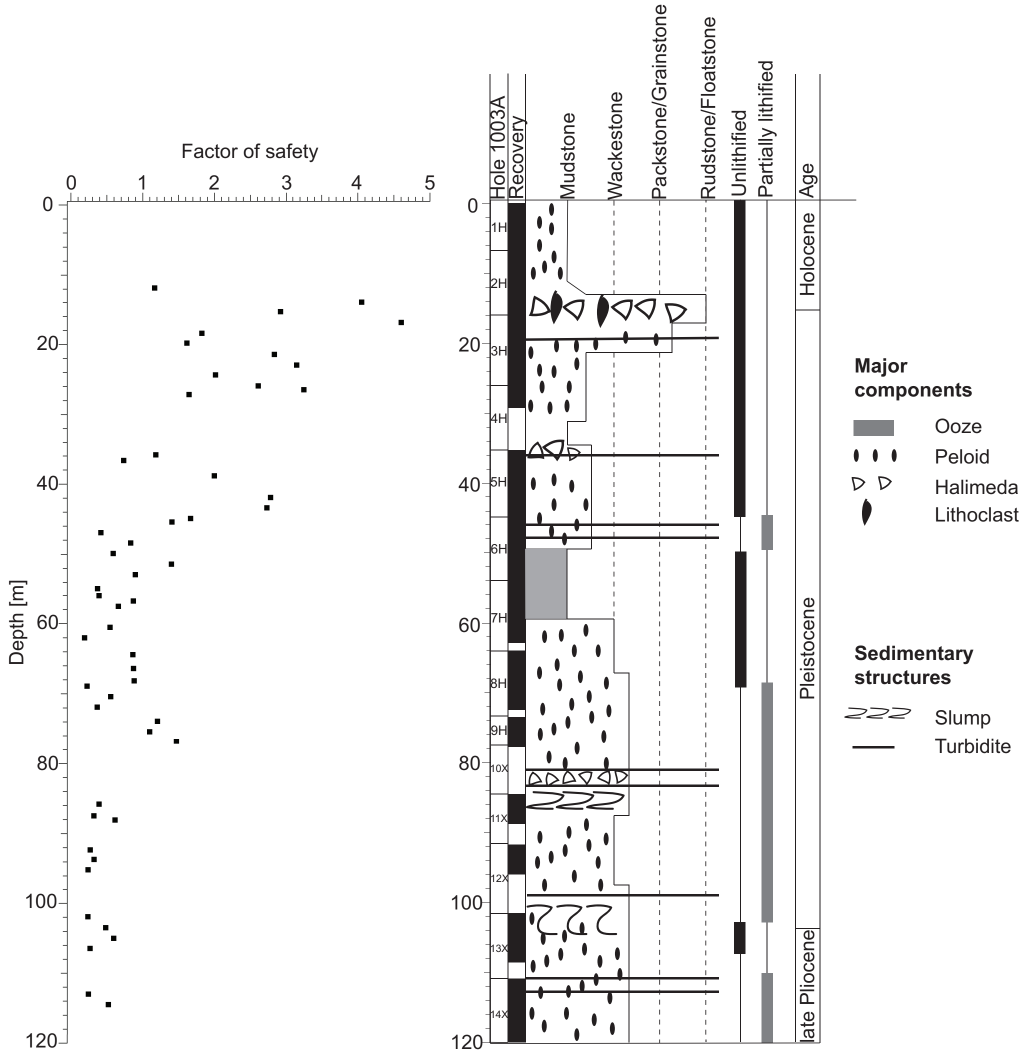

Figure S2: Peak wave height maps for all the simulations. Units are in meters. (Maps were created in MATLAB r2014a using GEBCO grids, http://www.gebco.net).

a) Partial landslide 20 m/s and 35.8 m/s;

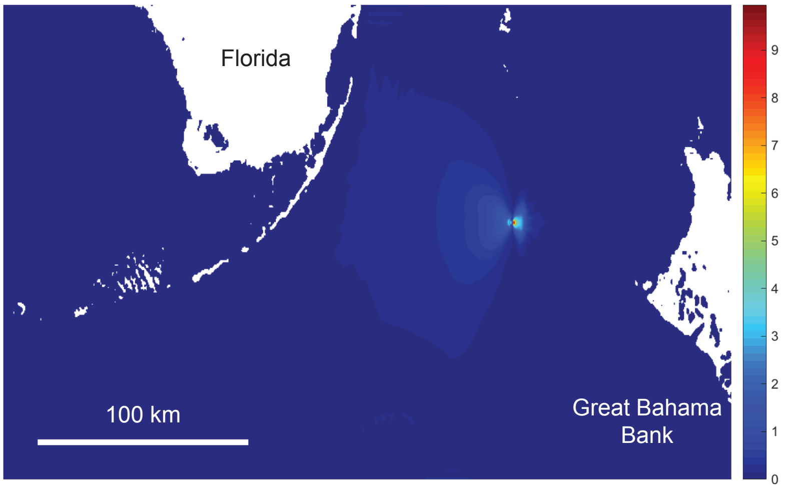

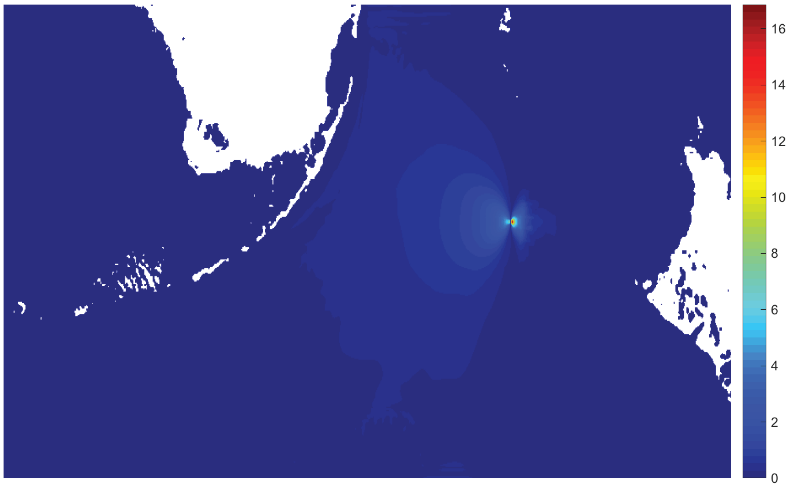

b) Single landslide 20 m/s and 38.7 m/s;

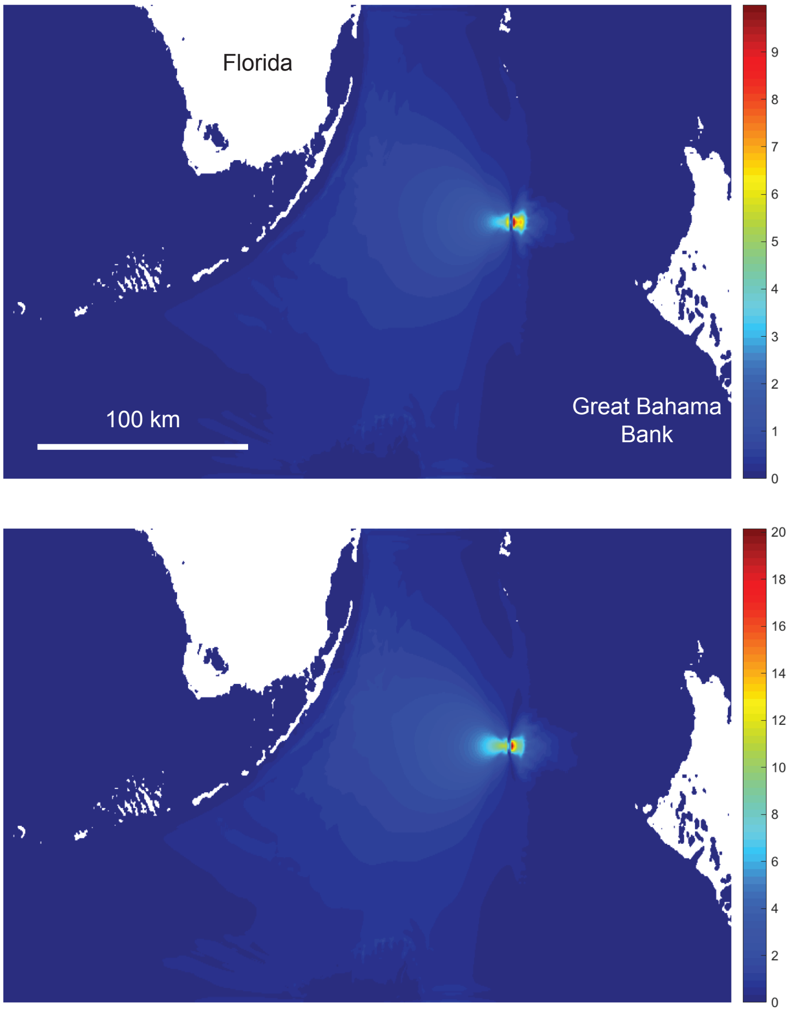

c) Potential future landslide 20 m/s and 48.32 m/s;

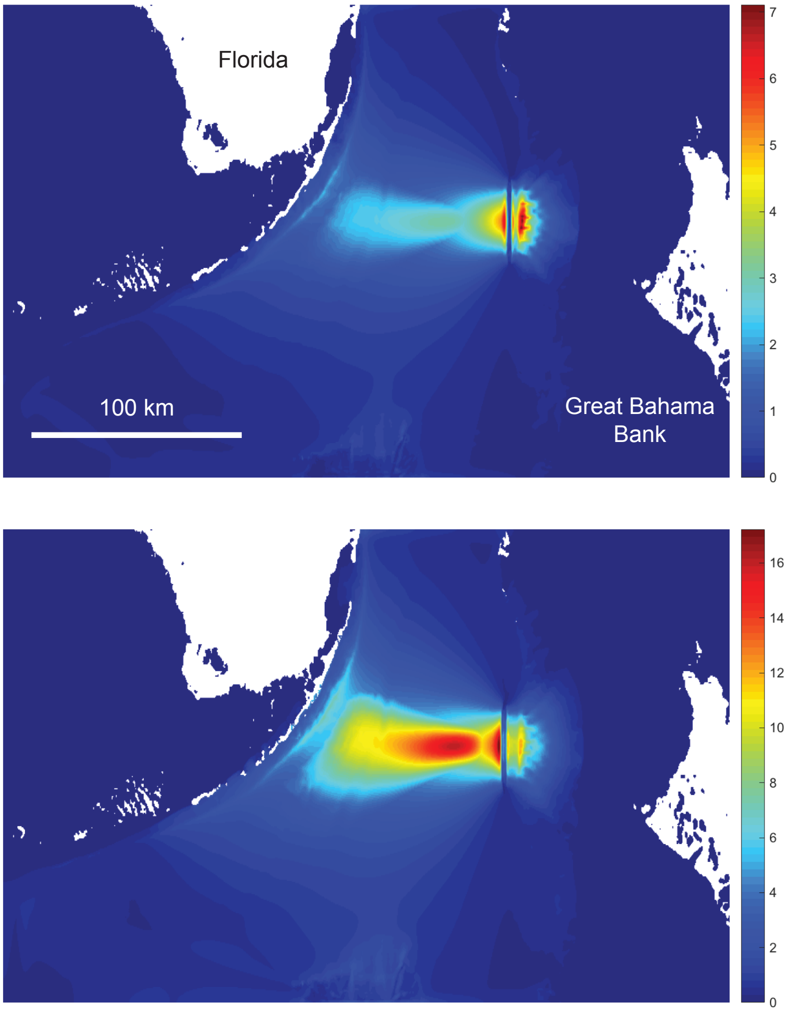

d) Margin collapse 20 m/s and 60.25 m/s;

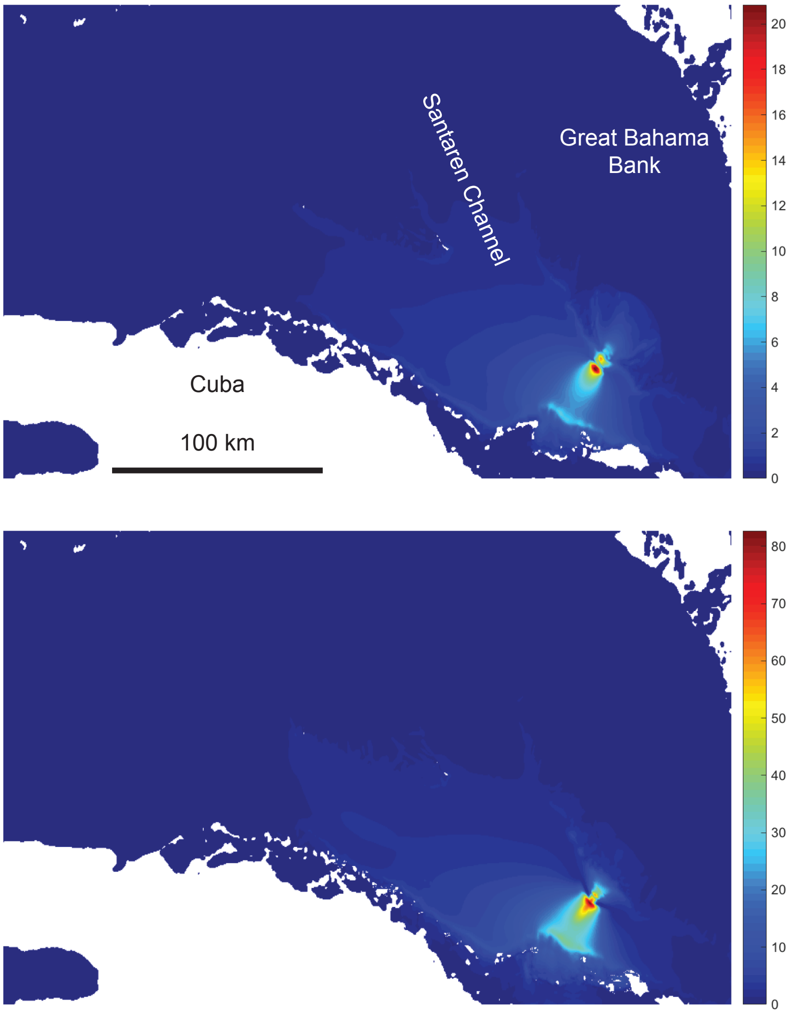

Supplement: Supplementary Information [file srep35925-s5.pdf]
